# Skeletal Muscle Redox Signaling in Health and Disease: From Molecular Mechanisms to Therapeutic Exercise Strategies

**DOI:** 10.3390/antiox15060678

**Published:** 2026-05-28

**Authors:** Hyeong Rok Yun, Manish Kumar Singh, Sunhee Han, Jyotsna S. Ranbhise, Hanjoon Seo, Sung Soo Kim, Insug Kang

**Affiliations:** 1 Department of Biochemistry and Molecular Biology, School of Medicine, Kyung Hee University, Seoul 02447, Republic of Korea; foryou018@naver.com (H.R.Y.);; 2 Biomedical Science Institute, Kyung Hee University, Seoul 02447, Republic of Korea; 3 Department of Biomedical Science, Graduate School, Kyung Hee University, Seoul 02447, Republic of Korea

**Keywords:** skeletal muscle, redox signaling, reactive oxygen species, NADPH oxidase, exercise training

## Abstract

Skeletal muscle plasticity is modulated by a delicate equilibrium between reactive oxygen species (ROS)-mediated signaling and oxidative distress. Although excessive oxidant accumulation impairs excitation–contraction coupling, accelerates fatigue, and contributes to muscle dysfunction, transient and compartmentalized ROS signals are now recognized as important modulators of mitochondrial biogenesis, metabolic remodeling, proteostasis, and tissue repair processes after contractile stress. This review synthesizes the biphasic nature of redox biology in exercise physiology, interpreting this duality through the paradigm of hormesis. We discuss modality-specific redox responses associated with endurance, resistance and high-intensity interval training, emphasizing that adaptive outcomes depend not on global redox shifts, but on spatiotemporally confined signaling cascades within specific nanodomains. Furthermore, we evaluate the controversial role of antioxidant supplementation, highlighting evidence that high-dose or poorly timed antioxidant intake attenuates specific exercise-induced adaptive responses. We further discuss how aging and chronic disease narrow the adaptive redox window by impairing mitochondrial quality control, inflammatory resolution, and recovery capacity. This paradigm supports a precision exercise strategy in which training modality, intensity, recovery, and nutritional interventions are aligned to preserve adaptive redox signaling while avoiding cumulative oxidative injury.

## 1. Introduction

Skeletal muscle is a highly plastic tissue that undergoes extensive structural, metabolic, and functional remodeling in response to repeated contractile stimuli. These adaptations include changes in mitochondrial density, substrate utilization, excitation–contraction coupling, capillary supply, and protein turnover [1,2,3,4]. However, the same contractile stimuli that promote adaptation can also impose substantial mechanical and metabolic stress, particularly during high-intensity exercise, unaccustomed eccentric loading, or prolonged endurance activity [5,6,7]. The nature of exercise-induced damage is modality-specific and spatially distinct. Resistance exercise primarily imposes mechanical strain and structural perturbations, including sarcomeric disruption such as Z-line streaming. These mechanical stresses may activate mechanosensitive redox pathways, including NOX2-associated X-ROS signaling, although Z-line streaming itself should be interpreted as a structural marker of damage rather than a direct upstream trigger of NOX2 activation [8,9,10]. In contrast, endurance exercise is characterized by metabolic stress and mitochondrial perturbations, where redox signals primarily drive mitochondrial quality control and mitophagy [11,12]. Thus, muscle damage should be defined by its specific structural or metabolic context rather than as a uniform phenomenon. However, contemporary evidence identifies physiologically generated reactive oxygen species (ROS) as integral signaling mediators essential for contractile force and adaptive remodeling [13]. Conversely, excessive or dysregulated oxidant production leads to oxidative distress. During oxidative distress, ROS and reactive nitrogen species (RNS) induce indiscriminate oxidative modifications to proteins and lipids, disrupt sarcoplasmic reticulum (SR) calcium handling, and impair excitation–contraction coupling, ultimately compromising contractile function [14,15,16,17]. Consequently, this biphasic nature underscores the pivotal role of redox biology in establishing exercise as a potent non-pharmacological tool for metabolic health. Critical challenges remain in defining the optimal type of exercise, exercise intensity and exercise volume to maximize adaptive signaling while mitigating oxidative damage [18,19]. Additionally, the role of antioxidant supplementation remains controversial, particularly concerning its potential to interfere with physiological signaling pathways [20,21]. Elucidating these complexities is paramount for establishing evidence-based guidelines that span from therapeutic applications in sarcopenia and metabolic disease to elite athletics. This review examines the dual role of reactive oxygen and nitrogen species (RONS) in skeletal muscle adaptation, starting with the molecular basis of redox hormesis. We compare the oxidative profiles of endurance, resistance and interval training, and critically evaluate how antioxidant supplementation can disrupt physiological signaling.

## 2. Molecular Mechanisms of Redox Adaptation

### 2.1. Redox Hormesis and the Optimal Redox Window

Exercise-induced redox signaling follows a hormetic dose–response relationship. Within an adaptive range, transient ROS act as second messengers that activate mitochondrial biogenesis, antioxidant defense, proteostasis, and remodeling pathways [22,23]. Below this range, redox signaling may be insufficient to induce adaptation, whereas excessive or prolonged oxidant exposure promotes oxidative distress and functional impairment. Based on the established concept of redox hormesis, we use the term “optimal redox window” as an operational descriptor for the adaptive range in which transient ROS signals support exercise-induced remodeling, whereas insufficient or excessive oxidant exposure limits adaptation or promotes oxidative distress. Transient ROS generated during contraction may also modulate anabolic signaling pathways, including mTORC1-related protein synthesis, although the requirement and magnitude of this effect appear to depend on exercise modality, redox source, and cellular context [8]. Under this paradigm, moderate-intensity exercise (e.g., 40–60% VO_2_max or consistent training volume) drives adaptive plasticity, specifically through the induction of antioxidant defense systems and mitochondrial quality control mechanisms [24,25]. In contrast, excessive intensity or prolonged duration without adequate recovery precipitates cellular dysfunction. Consequently, this paradigm highlights the fallacy of indiscriminately minimizing oxidative stress, as completely abolishing ROS transients can blunt the essential signaling pathways required for long-term physiological adaptation [20,21]. In applied settings, recovery-related indices, including performance recovery, countermovement jump performance, perceived fatigue, and plasma albumin thiol oxidation status, may provide indirect information on training tolerance and exercise-induced redox [6,26,27,28,29]. Although these markers cannot define an optimal redox state, they may help detect incomplete recovery or excessive fatigue, thereby informing conservative adjustment of training load. Here, “redox signaling” refers to transient, compartmentalized and reversible post-translational modifications (PTMs) with functional consequences, whereas “oxidative distress” denotes sustained or widespread modifications associated with impaired function. This distinction is fundamental, as redox signaling is orchestrated by reversible PTMs specific to distinct sites, such as the oxidation of thiol groups on cysteine residues, rather than by indiscriminate shifts in the global cellular redox state [30,31]. Accordingly, localized and transient ROS generation can drive significant biological adaptations even in the absence of systemic oxidative damage. This paradigm therefore supports an individualized perspective on exercise responses [32,33]. The magnitude of the redox perturbation generated by identical mechanical workload is modulated by training status, circadian rhythm, nutritional background, and baseline inflammatory profile [34,35,36]. Consequently, the inconsistent findings across antioxidant trials are a natural reflection of this heterogeneity. Efficacy is dictated by a strict context dependency whereby success relies on sustaining redox homeostasis, whereas failure results from shifting the balance into pathological states of reductive or oxidative overload.

### 2.2. Sources and Compartmentalization of RONS in Skeletal Muscle

The generation of RONS during contractile activity is a complex phenomenon emanating from discrete subcellular compartments. Although mitochondria have traditionally been postulated as the canonical source of superoxide (O_2_^•−^) via electron leak within the electron transport chain (ETC), emerging evidence reveals a complex mechanistic framework in which mitochondrial ROS emission alone is quantitatively insufficient to account for the total oxidative flux induced by exercise [13,37,38]. Cytosolic sources, particularly the nicotinamide adenine dinucleotide phosphate (NADPH) oxidase (NOX) family of enzymes, function as regulated generators of reactive species. The NOX2 and NOX4 isoforms are widely implicated as critical modulators of mechanotransduction and metabolic regulation, operating independently of mitochondrial respiration [37,39,40,41]. In parallel, Nitric Oxide (NO) signaling is a cornerstone of the redox framework. nNOS-derived NO modulates vascular perfusion, metabolic control, and excitation–contraction coupling. The interaction between NO and ROS (forming RNS) acts as an integrated molecular switch; however, impaired NO bioavailability, common in aging, destabilizes this balance and impairs contractile performance [42,43]. A more comprehensive discussion of ROS-RNS interactions is therefore essential for understanding the limits of skeletal muscle performance [44]. Collectively, these findings compel a paradigm shift from the traditional notion of reactive species as indiscriminate metabolic waste to a contemporary understanding of skeletal muscle as a regulated source of targeted redox signaling. Oxidative stress during exercise is not a monolithic phenomenon. It is more accurately characterized as a dynamic integration of signaling cascades defined by their specific sources, subcellular compartmentalization and temporal kinetics [45,46]. Recent mechanistic advances have further refined this paradigm through the concept of redox “nanodomains,” defined by the spatial juxtaposition of discrete RONS sources and their cognate targets [47,48]. In contracting myofibers, NOX2 localized to the sarcolemma and transverse tubules (T-tubules) generates highly compartmentalized hydrogen peroxide (H_2_O_2_) signaling. This process, termed ‘X-ROS signaling’, links the mechanical stretch of the cytoskeleton directly to NOX2 activation via the microtubule network [49]. These signals modulate membrane excitability and metabolic pathways, independently of any overt shifts in the global cellular redox state [8,50,51]. In contrast, NOX4 exhibits constitutive activity localized to intracellular compartments, regulating basal redox homeostasis via tonic mechanisms that diverge fundamentally from the inducible activation of NOX2 [41,52]. This compartment-specific framework reconciles the apparent paradox wherein standardized exercise protocols elicit divergent systemic biomarkers of oxidative damage yet consistently drive robust adaptive signaling. Beyond the canonical NOX and mitochondrial machinery, the mobilization of ancillary oxidant generation is contingent upon physiological context and dictated by the magnitude of contractile stress [53,54]. Xanthine oxidoreductase, phospholipase A2–mediated pathways and uncoupled NOS may significantly amplify oxidant flux during transitions of hypoxia–reoxygenation, inflammation, or metabolic dysregulation [55,56,57,58]. Furthermore, peroxisomal oxidases and endoplasmic reticulum (ER) oxidoreductases contribute to the cellular H_2_O_2_ levels, particularly under states of lipid overload or proteostatic stress [59,60,61]. Collectively, these findings suggest that exercise induces a distinct RONS profile. This profile depends not only on the amount of oxidants produced but also on the specific chemical species, their location, and how far they diffuse to reach target molecules. Recent evidence identifies the mitochondria-associated membranes (MAMs) as a pivotal hub for redox signaling. These contact sites between the ER and mitochondria facilitate the bidirectional transfer of Ca^2+^ and ROS. Nanodomain formation at MAMs allows NOX4-derived H_2_O_2_ to regulate mitochondrial Ca^2+^ uptake via the inositol 1,4,5-trisphosphate receptor (IP3R)-glucose-regulated protein 75 (Grp75)-voltage-dependent anion channel (VDAC) complex. This architecture tunes bioenergetics while minimizing diffusion of oxidant signals into the broader cytosol [48,62,63,64,65]. This structural coupling explains how muscle fibers achieve rapid metabolic shifts during the onset of contraction.

### 2.3. Endogenous Antioxidant and Redox-Buffering Systems

To preserve homeostasis within a physiological redox window, skeletal muscle is equipped with an intricate network of enzymatic and non-enzymatic antioxidant systems. These defenses are coordinated to mitigate oxidative perturbations while safeguarding the fidelity of essential signaling pathways [13,53,66]. Physical training promotes the robust upregulation of primary antioxidant enzymes, including superoxide dismutases (SODs), catalase (CAT) and glutathione peroxidases (GPx). This adaptive response enhances the buffering capacity in trained muscle, allowing attenuation of oxidative perturbations at matched absolute workloads [67,68]. Notably, the antioxidant network operates as a compartmentalized rheostat controlling the amplitude, duration and localization of oxidative pulses. This mechanism ensures that reactive species transduce adaptive signals while averting irreversible macromolecular damage [32,66,69]. Furthermore, redox regulation exhibits pronounced fiber type specificity. Slow oxidative and fast glycolytic fibers are characterized by distinct mitochondrial densities and antioxidant profiles. This intrinsic heterogeneity mandates a fiber-type-specific perspective, particularly in aging. Type I fibers generally exhibit higher mitochondrial density and stronger basal antioxidant buffering, whereas Type II fibers are more susceptible to oxidative perturbation but are also more responsive to high-intensity and resistance-type stimuli. Thus, the preferential loss of Type II fibers during aging does not necessarily imply a simple decline in whole-muscle antioxidant capacity. Instead, it reduces the fiber pool available for robust mechanical, metabolic, and redox-sensitive remodeling. Consequently, aged muscle may retain basal antioxidant buffering in the remaining oxidative fibers but display a narrower adaptive range in response to vigorous or resistance-based exercise [19,70,71,72,73]. Incorporating this perspective is important for tailoring exercise protocols in clinical populations in which muscle fiber-type distribution and exercise responsiveness are substantially altered [33].

### 2.4. Redox Signaling in Physiological Adaptation

**Figure 1:**
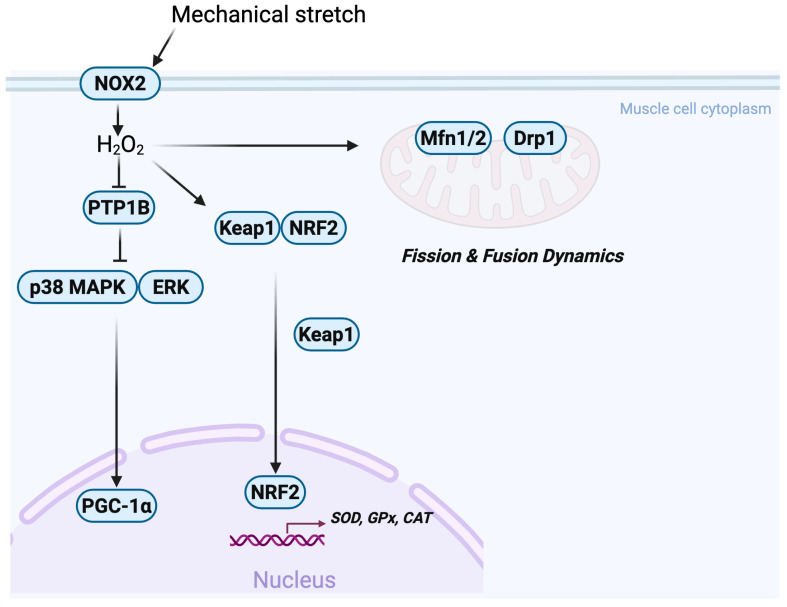
Compartmentalized redox signaling pathways in skeletal muscle adaptation. Mechanical stretch activates sarcolemmal NOX2, generating localized H_2_O_2_. This oxidant flux coordinates three major adaptive responses. First, H_2_O_2_ transiently inactivates PTP1B, thereby sustaining the activation of p38 MAPK and extracellular signal-regulated kinase (ERK) to promote PGC-1α-mediated mitochondrial biogenesis. Second, H_2_O_2_ disrupts the Keap1-NRF2 complex, allowing NRF2 to translocate to the nucleus and upregulate antioxidant enzymes (SOD, GPx, CAT). Third, localized ROS modulate mitochondrial fission and fusion dynamics through the regulation of Mfn1/2 and Drp1, ensuring organelle quality control. (Figure created in Biorender. Hyeong Rok Yun. (2026) https://app.biorender.com/illustrations/canvas-beta/69808bb5edbcca9160a567ec, accessed on 25 May 2026).

The integration of redox biology and exercise physiology highlights the pivotal role of RONS in modulating the signaling networks that regulate skeletal muscle remodeling. Current paradigms emphasize that RONS act as critical signaling transducers, orchestrating the activity of kinases, phosphatases and transcriptional machinery essential for mitochondrial adaptation and metabolic flexibility [13,74,75]. This conceptual shift highlights that RONS function as indispensable mediators of exercise adaptation, rather than merely as deleterious byproducts of metabolism. This perspective is supported by evidence indicating that the suppression of oxidative signaling attenuates specific training-induced adaptations in humans [13,20,76]. Furthermore, mechanistic studies indicate that NOX-dependent ROS generation is integral to exercise-mediated metabolic regulation, suggesting that specific ROS sources function as upstream regulators rather than passive byproducts of contractile activity [9,37,77]. At the molecular level, the dominant signaling paradigm is increasingly characterized by reversible and residue-specific cysteine modifications rather than indiscriminate oxidation [30]. Transient H_2_O_2_ levels are transduced through redox relay systems, particularly peroxiredoxins and thioredoxin networks. These systems convert short-lived oxidant signals into stable, selective thiol modifications on kinases, phosphatases, and transcriptional regulators [78,79]. The sarcoplasmic reticulum Ca^2+^-ATPase (SERCA) undergoes reversible S-glutathionylation at Cys674, which preserves Ca^2+^ uptake activity under physiological stress but is irreversibly sulfonated during chronic oxidative distress [80,81]. Similarly, Ryanodine Receptor 1 (RyR1) possesses highly reactive cysteine residues (e.g., Cys3635) that function as a physiological gain sensor for calmodulin binding and channel opening [82,83]. These redox-sensitive mechanisms demonstrate that adaptation extends beyond transcriptional regulation to include the acute post-translational tuning of the excitation–contraction machinery. In parallel, redox-dependent modulation regulates key signaling enzymes. For instance, the oxidation of the catalytic cysteine residue in phosphatases such as protein tyrosine phosphatase 1B (PTP1B) induces transient inactivation. This inhibition preserves the phosphorylation status of upstream kinases, effectively lowering the activation threshold for anabolic signaling cascades [84]. This framework elucidates how small and spatially restricted oxidant transients can effectively reprogram gene expression despite minimal changes in bulk redox couples. Multiple redox-sensitive nodes integrate contractile activity with long-term plasticity. ROS potentiate endurance adaptation by converging on AMP-activated protein kinase (AMPK) and p38 mitogen-activated protein kinase (p38 MAPK) signaling, thereby promoting peroxisome proliferator-activated receptor-γ coactivator-1α (PGC-1α)-dependent mitochondrial biogenesis and the upregulation of oxidative enzymes [13,38]. In parallel, Kelch-like ECH-associated protein 1 (Keap1) and nuclear factor erythroid 2-related factor 2 (NRF2) signaling function as a pivotal transcriptional hub that translates repeated oxidant bursts into the robust induction of antioxidant defenses and proteostatic networks, effectively converting transient redox stress into enhanced physiological resilience [85,86]. Furthermore, this redox-centric paradigm extends to mitochondrial quality control. Exercise-mediated mitophagy and mitochondrial proteostasis pathways, including mitochondrial unfolded protein response (UPRmt)-related programs, are now recognized as adaptive effectors that can be tuned by redox status and energetic stress, thereby linking acute oxidant signaling to the preservation of organelle integrity [87,88,89,90]. Specifically, redox-sensitive modifications dictate mitochondrial network architecture by regulating key GTPases such as dynamin-related protein 1 (Drp1) and mitofusin 1 and 2 (Mfn1/2). This regulation ensures the segregation of damaged organelles for subsequent mitophagy, thereby maintaining the quality of the mitochondrial network [91,92,93]. An integrated model of these compartmented redox signaling cascades, highlighting the coordinated regulation of anabolic pathways, antioxidant defense, and mitochondrial dynamics, is illustrated in Figure 1. Redox regulation also provides a direct mechanistic link between molecular signaling and contractile performance. Within the adaptive redox range, reversible thiol modifications of Ca^2+^-handling proteins, including RyR1 and SERCA, can fine-tune Ca^2+^ release, Ca^2+^ reuptake, and force generation. Conversely, excessive or sustained oxidative modification promotes SR Ca^2+^ leak, impaired Ca^2+^ reuptake, and excitation–contraction (E-C) uncoupling, thereby contributing to fatigue, weakness, and maladaptive responses during overreaching [15,80,94,95,96,97,98]. Table 1 provides a comparative overview of the evidence derived from mechanistic cell/animal models versus human clinical trials, highlighting the current limitations in translational spatial resolution.

## 3. Exercise-Induced Redox Responses and Training Paradigms

### 3.1. Exercise Modality-Specific Redox Landscapes

Endurance exercise is fundamentally characterized by sustained metabolic demand and elevated oxygen flux. This physiological state engenders a prolonged, low-to-moderate oxidant environment [13,99]. Chronic endurance training promotes mitochondrial biogenesis and enhanced oxidative capacity [100]. ROS signaling is increasingly recognized as an integral regulator of the transcriptional and translational machinery driving these adaptations. The physiological phenotype of endurance training is characterized by an improved capacity to modulate oxidant flux. This is accomplished not by abrogation of ROS production, but through the optimization of redox buffering capacity and resilience mechanisms [101,102]. Conversely, resistance exercise elicits a distinct redox profile, predominantly defined by high-magnitude mechanical strain and subsequent drive for tissue remodeling [73,103]. In this modality, the oxidative signal may arise from mechanotransduction, transient ischemia–reperfusion within contracting fibers and immune cell infiltration during the recovery phase [104]. Under these conditions, a moderate inflammatory and oxidative response is not necessarily pathological, but may support tissue repair, remodeling, and the recovery environment required for effective adaptation [105,106,107]. Maladaptation following resistance exercise arises when the magnitude of the mechanical and oxidative stimulus exceeds the individual’s recovery capacity, a phenomenon often observed in states of chronic overreaching. Sustained elevations in ROS have been shown to antagonize the mTORC1 signaling pathway, the primary driver of muscle protein synthesis, while simultaneously inducing oxidative damage to the DNA and protein of muscle stem cells, which compromises their proliferative capacity [108,109,110]. These mechanisms may contribute to an anabolic-resistant phenotype, particularly when excessive loading is combined with insufficient recovery, chronic inflammation, aging, or metabolic dysfunction [111,112].

High-intensity interval training (HIIT) and sprint interval training (SIT) elicit acute, intermittent surges of metabolic stress, characterized by rapid oscillations between peak energetic demand and partial recovery. These metabolic fluctuations trigger potent signaling cascades that induce adaptation in a highly time-efficient manner, thereby underpinning the efficacy of HIIT in improving cardiorespiratory fitness [74,113,114,115]. However, the intensity of these oxidative insults may overwhelm the buffering capacity in individuals with compromised recovery mechanisms or excessive baseline oxidative stress [116,117]. Consequently, while HIIT constitutes a potent therapeutic stimulus, it requires careful titration, particularly in aging populations or patients with underlying chronic pathologies. In these vulnerable populations, the therapeutic window or hormetic range between beneficial signaling and deleterious stress is markedly constricted [98,118,119].

### 3.2. Muscle Damage, Inflammation, Recovery and Overload

**Figure 2:**
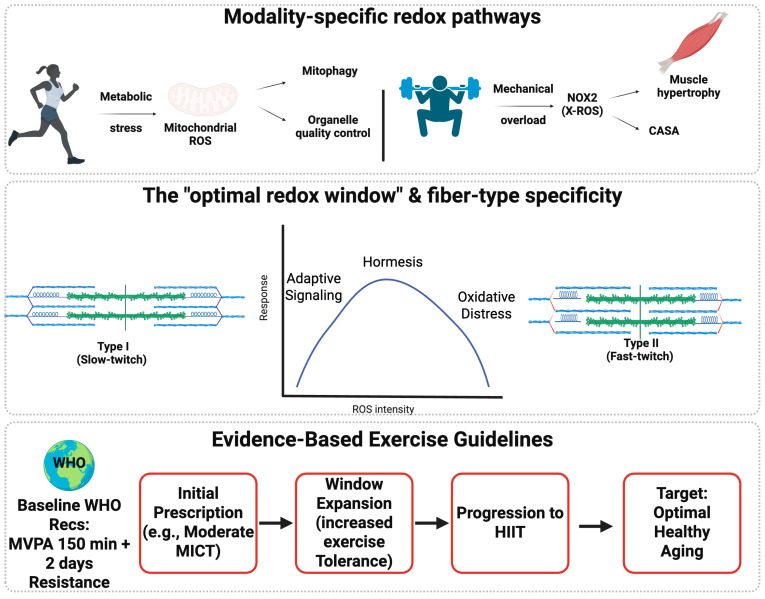
Compartmentalized redox signaling in skeletal muscle adaptation and translational guidelines. This holistic schematic delineates skeletal muscle redox signaling from basic molecular mechanisms to personalized exercise medicine based on evidence-based guidelines. **Top Panel** (Modality-Specific Pathways): Aerobic exercise-induced metabolic stress is detailed, leading to mitochondrial ROS and mitophagy. Conversely, resistance exercise-induced mechanical overload activates the NOX2 pathway, driving muscle hypertrophy and CASA. **Middle Panel** (Optimal Redox Window & Fiber-Type Specificity): The central Hormesis curve demonstrates the optimal ROS range for adaptive signaling versus distress, comparing a wider window in Type I sarcomeres to a narrower, fragile window in aging-susceptible Type II sarcomeres. **Bottom Panel** (Translational Application): Building upon WHO Recommendations (MVPA 150 min + 2 days resistance), the flowchart details a structured progression from MICT for window expansion to HIIT for optimal healthy aging through targeted exercise medicine. (Figure created in Biorender. Hyeong Rok Yun. (2026) https://app.biorender.com/illustrations/canvas-beta/69c92b8cc136a810a91e8d0b, accessed on 25 May 2026).

Unaccustomed eccentric contractions frequently induce delayed onset muscle soreness (DOMS) and transient functional impairment. These mechanical perturbations are intimately coupled with a robust inflammatory cascade. This inflammatory cascade is characterized by a surge in oxidant generation, emanating from both infiltrating immune cells and mechanically strained myofibers [120,121]. During physiological recovery, ROS transients are better understood as permissive and modulatory signals rather than direct drivers of protein synthesis. They help coordinate proteolytic clearance, inflammatory resolution, satellite cell activation, and remodeling pathways required for effective regeneration. They orchestrate the removal of damaged proteins via the ubiquitin-proteasome system and trigger satellite cell activation through p38 MAPK-dependent signaling, thereby establishing the necessary cellular environment for structural rebuilding [23,122,123,124]. This adaptive phenomenon is collectively termed the repeated bout effect (RBE) [125,126]. Mechanistically, the NOD-like receptor family pyrin domain containing 3 (NLRP3) inflammasome operates as a critical checkpoint in this process, primed by sensing of mitochondrial damage-associated molecular patterns (DAMPs) and mitochondrial ROS (mtROS) [127,128,129]. Specifically, controlled NLRP3 activation mediates the release of interleukin-1 beta (IL-1β) and interleukin-18 (IL-18) to orchestrate satellite cell proliferation and tissue clearance [130,131]. Recent single-cell transcriptomic studies reveal that the shift from pro-inflammatory classically activated macrophages (M1) to anti-inflammatory alternatively activated macrophages (M2) is metabolically gated by intercellular ROS for phagocytic activity. The subsequent transition to a reparative M2 phenotype requires a metabolic switch to oxidative phosphorylation and fatty acid oxidation, a process strictly regulated by NRF2-mediated redox buffering [132,133,134,135,136]. Thus, muscle recovery is fundamentally an immunometabolic event orchestrated by redox timing. Conversely, sustained or dysregulated inflammasome signaling exacerbates oxidative injury, compromises contractile function and impedes tissue regeneration. Such mechanistic insight is pivotal for the optimization of exercise prescriptions [137,138]. Attrition in exercise interventions is frequently caused by intolerance to soreness and fatigue, rather than by actual pathological injury [139]. Recognizing that controlled inflammatory and redox signaling can support remodeling helps distinguish expected post-exercise discomfort from signs of maladaptation or injury. However, soreness should not be regarded as a necessary marker of effective training, because adaptation can occur in the absence of substantial pain [13,125]. This interpretation remains valid, however, only if training progression and recovery are meticulously titrated. Conversely, persistent fatigue and performance deterioration suggest that cumulative allostatic load has overwhelmed systemic recovery capacity [140]. In this state, chronic oxidative stress and inflammation shift from adaptive signals to drivers of maladaptive dysfunction [112,117]. The divergent redox signatures and human evidence levels for these training modalities are synthesized in Table 2. Literature investigating high training loads and insufficient recovery frequently attributes maladaptation to a fundamental mismatch between the magnitude of applied stress and the individual’s adaptive capacity [112]. Importantly, the resolution of exercise-induced damage depends not only on inflammatory signaling but also on intracellular protein quality control systems. Redox signaling directly interfaces with cytoskeletal repair pathways, such as Chaperone-Assisted Selective Autophagy (CASA) [141]. ROS-mediated modifications of these repair machineries are essential for maintaining myofibrillar structural integrity and enabling effective regeneration following high-magnitude mechanical stress [142]. Consequently, the management of oxidative stress transcends molecular targets, necessitating a holistic strategy that integrates training architecture, sleep hygiene, nutritional support and periodization [117,143,144]. From the perspective of healthspan, sustainable exercise regimens must deliver a sufficient stimulus for adaptation while minimizing the risk of chronic inflammation and cumulative oxidative injury. Figure 2 integrates these concepts into a translational paradigm in which modality-specific redox pathways, fiber-type-dependent vulnerability, and progressive exercise prescription converge to define a precision exercise strategy.

## 4. Translational Aspects

Emerging research demonstrates that skeletal muscle ROS signaling extends beyond local adaptation, influencing systemic physiology via redox-regulated exerkine secretion. Exercise-induced ROS have been shown to modulate the biogenesis and cargo loading of extracellular vesicles (EVs), which mediate crosstalk with adipose tissue and the liver [24,145,146]. This suggests that the systemic benefits of exercise, such as improved insulin sensitivity, are partly dependent on the fidelity of muscle redox signaling [22].

### 4.1. Antioxidant Supplementation and Redox Adaptation

The theoretical rationale for exogenous antioxidant supplementation suggests that the pharmacologic scavenging of ROS will attenuate exercise-induced oxidative stress and accelerate functional recovery. However, human training studies do not consistently support the assumption that antioxidant supplementation improves recovery or adaptation. Instead, several trials indicate that high-dose antioxidant supplementation, particularly with vitamins C and E, can blunt selected exercise-induced adaptations, including mitochondrial biogenesis-related signaling and fiber-type-specific proteomic remodeling [20,147]. These findings support the concept that exercise-induced ROS are not merely cytotoxic byproducts, but context-dependent signaling molecules required for selected adaptive responses. Furthermore, human training studies indicate that supraphysiological doses of vitamins C and E may impede key cellular adaptations to endurance training, including the upregulation of regulatory proteins related to mitochondrial biogenesis [148]. However, these findings should not be extrapolated to imply a universally deleterious effect of antioxidant supplementation. Instead, these findings suggest that removing or dampening the oxidative stimulus via high-dose supplementation may abrogate the signals necessary for hormetic adaptation in specific contexts. This distinction is particularly relevant when ingestion is temporally coupled with the exercise stimulus. Such a perspective reveals the concept of hormesis and the consensus that oxidants serve as essential signaling transducers, orchestrating tissue remodeling [149,150,151]. A stratified approach conceptualizes nutritional interventions as contingent upon physiological status. Accordingly, targeted antioxidant support may be preferentially indicated for populations with documented deficiencies, clinical indications, or pathologically elevated oxidative stress [152,153,154]. In contrast, healthy individuals aiming to maximize physiological adaptations derive superior benefit from dietary sufficiency rather than supplementation. Consequently, indiscriminate high-dose supplementation within the peri-training window is discouraged [144,155]. Crucially, nutritional modulation transcends isolated supplementation. Whole-food matrices and strategic macronutrient timing can augment recovery and training quality while preserving essential redox signaling pathways.

### 4.2. Aging and Chronic Disease

Biological aging is characterized by a progressive decline in mitochondrial function, compromised redox buffering capacity and persistent inflammation [156,157]. This baseline oxidative stress is further compounded by the presence of various comorbidities [158]. Nevertheless, exercise emerges as a potent non-pharmacological intervention capable of preserving functional capacity, bolstering metabolic health and attenuating disease risk [159]. Notably, geriatric cohorts and clinical populations frequently exhibit a perturbed redox status [160,161]. Consequently, a fixed absolute exercise stimulus may elicit a disproportionately amplified physiological strain in these cohorts relative to healthy controls. These findings should not be interpreted as advocating for the complete avoidance of oxidative stress. Instead, they emphasize the necessity of tailored program design and gradual load titration. The therapeutic goal is to elicit a hormetic redox stimulus without precipitating maladaptive inflammation or unremitting oxidative damage [23]. Mechanistic evidence implicating NOX pathways in adaptive responses suggests that ROS generation remains indispensable for beneficial remodeling [8,22,162]. In aging and chronic disease, the core architecture of ROS-dependent signaling is not abolished, but its functional dynamic range is reduced. Impaired mitochondrial quality control, neuromuscular junction instability, chronic low-grade inflammation, and delayed inflammatory resolution collectively narrow the range in which ROS can act as adaptive signals [163,164,165]. Specifically, age-related denervation and impaired neuromuscular junction (NMJ) integrity destabilize mitochondrial redox homeostasis, thereby eroding the fidelity of redox signaling relays [12,24,166]. As a result, aged or diseased muscle may display a paradoxical phenotype: higher basal oxidant production but weaker adaptive signal transduction in response to exercise. In parallel, declines in nicotinamide adenine dinucleotide (NAD^+^) availability and sirtuin signaling, together with impaired mitophagic turnover, facilitate the accumulation of dysfunctional organelles, elevating the basal state to one of persistent stress [167,168,169]. A pivotal emerging paradigm suggests that the therapeutic efficacy of exercise under such pathological conditions hinges less on indiscriminate ROS scavenging, and more on reinstating redox sensitivity via mitochondrial proteostasis and organelle turnover [12,89,170]. Consequently, the exercise-induced orchestration of UPRmt and mitophagy, coupled with mitohormetic signaling, has emerged as a central mechanistic paradigm underpinning improved muscle function despite a high baseline oxidative stress. This perspective suggests that therapeutic strategies targeting recovery capacity, mitochondrial turnover and inflammatory resolution may expand the therapeutic redox window and improve tolerance to higher-intensity prescriptions [23,89,171,172]. In prescribing exercise to extend health span, the overarching principle is physiological alignment [173]. Aligned with WHO global recommendations, precision exercise prescription must integrate both endurance and resistance modalities while respecting individual recovery kinetics. For deconditioned or clinical cohorts, an initial phase of moderate-intensity continuous training (MICT) is advised to expand the redox window by bolstering antioxidant defenses. This physiological foundation allows for the subsequent and safe introduction of High-Intensity Interval Training (HIIT) or resistance protocols, maximizing adaptive signaling while minimizing the risk of impaired inflammatory resolution [173,174,175,176]. This distinction is paramount in clinical populations. In such settings, the primary objective is often the optimization of functional status and quality of life rather than the attainment of maximal performance [177,178].

### 4.3. Exercise Protocols for Health: Practical Translational Principles

The optimization of adaptive outcomes necessitates the strategic alignment of exercise modalities with specific redox-mediated pathways. To promote mitochondrial biogenesis and oxidative capacity, moderate-intensity continuous training (MICT, 50–70% VO_2_max) is particularly efficacious in maintaining the sustained, low-magnitude ROS flux required for chronic signaling. Conversely, rapid metabolic reprogramming and the robust upregulation of antioxidant defenses are more effectively triggered by high-intensity interval training (HIIT), which induces potent, intermittent bursts of oxidative stress. For skeletal muscle hypertrophy, resistance training is indispensable to engage mechanical-stretch-dependent NOX2 activation, thereby driving the downstream anabolic cascades essential for myofibrillar remodeling [159,173]. These adaptations enhance redox regulation and cellular resilience over time [13]. In contrast, among deconditioned cohorts, the initiation of training often triggers a marked oxidative and inflammatory perturbation [179,180]. In sedentary and clinical populations, constrained redox-buffering capacity and impaired repair responsiveness narrow the adaptive range, making gradual load titration essential for preserving hormetic signaling while avoiding maladaptive oxidative distress [101,181]. This strategy permits endogenous defense mechanisms and remodeling processes to accommodate the training stimulus. Although the therapeutic value of exercise in aging and chronic disease is well established, carefully designed exercise protocols remain essential [182,183]. Such individualized programming requires careful calibration of exercise intensity, volume, frequency, and recovery intervals. It aligns training stress with recovery kinetics and redox buffering capacity. This alignment reduces cumulative distress while preserving adaptive signaling across repeated sessions. The implementation of exercise-as-medicine paradigms mandates the holistic assessment of functional reserve, systemic inflammatory profile and recovery kinetics [33,184,185]. Universal physiological imperatives transcend specific training modalities, encompassing the calibration of initial loads to individual tolerance, systemic load titration and the strategic incorporation of restorative intervals [186,187,188]. The concurrent integration of endurance and resistance modalities supports metabolic homeostasis while conserving muscle mass and contractile integrity [189]. This strategy is particularly relevant for the prevention of frailty [190].

## 5. Methodological Considerations and Future Directions

A major challenge in this field remains the inherent complexity of RONS. These species exhibit a transient nature, spatial compartmentalization and chemical heterogeneity. Generalized assertions that exercise increases oxidative stress offer limited insight unless the temporal dynamics, specific context and functional consequences are elucidated. Contradictory findings within the literature are frequently attributable to heterogeneity in exercise protocols, participant characteristics, and sampling timing [22,191,192,193]. Furthermore, the distinction between physiological oxidative signaling and pathological oxidative damage is often ambiguous. A major reason for this ambiguity is methodological. Traditional methods often measure accumulated oxidation products or nonspecific probe oxidation, thereby masking the rapid, compartmentalized transients that regulate signaling [193,194,195]. A key trend is shifting from endpoint markers to spatiotemporally resolved measurements. Genetically encoded biosensors, including HyPer (a H_2_O_2_ sensor) and roGFP-based redox reporter variants, allow for real-time monitoring with high specificity [196,197]. Recent methodological reviews highlight the need for in vivo imaging to quantify organelle-specific ROS kinetics, rather than inferring flux from systemic biomarkers [193,198]. Concurrently, redox proteomics has advanced from simply cataloging oxidized proteins to identifying residue-specific cysteine oxidation states, such as sulfenylation and other reversible thiol modifications. The emerging cysteine redox proteoform framework suggests that proteins can adopt multiple functional states [199,200]. Consequently, adaptation or dysfunction may be driven by shifts in the distribution of these states across contractile and metabolic networks. Integrating these approaches with training interventions and functional endpoints, including recovery kinetics and performance metrics, is likely to define the next wave of high-impact translational studies in exercise redox biology. Quantifying oxidant magnitude in isolation provides limited insight [201]. Future studies must establish how specific fluxes affect force production, fatigue resistance and long-term adaptation. Additionally, the modulatory role of biological sex, age and disease status in these dynamics warrants further investigation [4,33]. Achieving mechanistic clarity depends on integrating human trials with experimental models that isolate causal pathways while preserving physiological relevance [22]. Translational work should focus on addressing actionable questions regarding training design and clinical exercise prescription. Research aims to establish exercise protocols that optimize beneficial redox signaling while avoiding prolonged physiological impairments. Moreover, nutritional strategies must be optimized to support rather than suppress these adaptive signals [20]. This translational perspective is consistent with the paradigm of exercise as medicine and interventions targeting sedentary lifestyles.

## 6. Conclusions

Exercise-induced redox biology is best understood as a hormetic and compartmentalized signaling system rather than a simple balance between oxidant production and antioxidant defense. Transient, spatially restricted ROS signals support mitochondrial remodeling, proteostasis, inflammatory resolution, and contractile adaptation, whereas sustained or poorly resolved oxidative distress promotes fatigue, impaired recovery, and maladaptation. Consequently, translational strategies must move beyond indiscriminate antioxidant supplementation, which may compromise essential signaling pathways, toward personalized exercise prescriptions that respect individual recovery kinetics. Future advances depend on spatiotemporally resolved methodologies to accurately define the optimal redox window. Integrating compartment-specific redox biology with individualized training design may therefore provide a mechanistic foundation for precision exercise medicine, particularly in aging and chronic disease states where the adaptive redox window is narrowed.

## Figures and Tables

**Table 1:** Distinction of Redox signaling Evidence: Mechanistic Models vs. Human Exercise Studies.

Feature	Mechanistic Models (Cell/Animal)	Human Exercise Studies
Source Identification	Direct: Uses Genetic KO/Inhibitor for NOX2, NOX4 and Mito.	Indirect: inferred from systemic markers (TBARS, PC) or biopsy analysis
Spatial Resolution	High: Subcellular nanodomains (Mito vs. SR) via real-time sensors.	Low: Global muscle tissue or systemic redox status; limited subcellular data.
Causal Evidence	Definitive: Proves ROS as a necessity for adaptation via loss-of-function.	Correlative: Observational associations between redox flux and performance.
Temporal Dynamics	Real-time: Monitoring ROS transients during active contraction.	Snapshot: Biopsies collected at discrete time points post-exercise.

**Table 2:** Comparative Analysis of Exercise Modalities and Redox Signaling Outcomes in Humans.

Exercise Modality	Predominant Redox Source	Candidate Adaptive Pathways	Strength of Evidence (Humans)
Endurance (MICT)	Mitochondrial (Complex I/III), NOX4	PGC-1α, AMPK, SIRT1, NRF2	High: Extensive longitudinal training data.
Resistance	NOX2 (X-ROS), Mechanical-stretch.	mTORC1, Satellite cells, Myogenin	Moderate: Mechanistic links largely inferred.
HIIT/SIT	Mixed (Mito-ROS & NOX flux)	PGC-1α, NRF2, Mitochondrial biogenesis	Moderate to High: Emerging clinical evidence.
Eccentric-heavy	NOX2, Inflammatory cells	NF-κB, Satellite cell proliferation	Moderate: Clear post-exercise oxidative markers.

## Data Availability

No new data were created or analyzed in this study. Data sharing is not applicable to this article.

## Abbreviations

AMPK	AMP-activated protein kinase
CAT	Catalase
DAMPs	Damage-associated molecular patterns
DOMS	Delayed onset muscle soreness
Drp1	Dynamin-related protein 1
E-C	Excitation–contraction
ER	Endoplasmic reticulum
ERK	Extracellular signal-regulated kinase
ETC	Electron transport chain
EVs	Extracellular vesicles
GPx	Glutathione peroxidases
Grp75	Glucose-regulated protein 75
H_2_O_2_	Hydrogen peroxide
HIIT	High-intensity interval training
IL-18	Interleukin-18
IL-1β	Interleukin-1 beta
IP3R	Inositol 1,4,5-trisphosphate receptor
Keap1	Kelch-like ECH-associated protein 1
M1	Classically activated macrophages
M2	Alternatively activated macrophages
MAMs	Mitochondria-associated membranes
Mfn1/2	Mitofusin 1 and 2
mtROS	Mitochondrial ROS
MVPA	Moderate-to-vigorous physical activity
NAD^+^	Nicotinamide adenine dinucleotide
NADPH	Nicotinamide adenine dinucleotide phosphate
NLRP3	NOD-like receptor family pyrin domain containing 3
NMJ	Neuromuscular junction
NOS	Nitric oxide synthase
NOX	NADPH oxidase
NRF2	Nuclear factor erythroid 2-related factor 2
p38 MAPK	p38 mitogen-activated protein kinase
PGC-1α	Peroxisome proliferator-activated receptor-γ coactivator-1α
PTMs	Post-translational modifications
PTP1B	Protein tyrosine phosphatase 1B
RBE	Repeated bout effect
RNS	Reactive nitrogen species
RONS	Reactive oxygen and nitrogen species
ROS	Reactive oxygen species
RyR1	Ryanodine receptor 1
SERCA	Sarcoplasmic reticulum Ca^2+^-ATPase
SIT	Sprint interval training
SODs	Superoxide dismutases
SR	Sarcoplasmic reticulum
TBARS	Thiobarbituric acid reactive substances
UPRmt	Mitochondrial unfolded protein response
VDAC	Voltage-dependent anion channel
